# DOES WORKFORCE ROLE IMPACT LEARNING IN NURSING HOME EDUCATION? EVIDENCE FROM THE CHATO NATIONAL TRIAL

**DOI:** 10.1093/geroni/igad104.1112

**Published:** 2023-12-21

**Authors:** Clarissa Shaw, Kristine Williams, Maria Hein, Yelena Perkhounkova, Carissa Coleman, Frances Yang

**Affiliations:** University of Iowa, Iowa City, Iowa, United States; University of Kansas Medical Center, Kansas City, Kansas, United States; University of Iowa, College of Nursing, Iowa City, Iowa, United States; University of Iowa, Iowa City, Iowa, United States; University of Kansas, Kansas City, Kansas, United States; University of Kansas, Kansas City, Kansas, United States

## Abstract

Nursing home residents rely on a variety of staff to meet their physical and psychosocial needs. Our communication intervention for nursing home staff—entitled CHATO—is currently being tested in a pragmatic clinical trial nationwide. All staff, regardless of role, are encouraged to participate to create a culture of person-centered communication. We analyzed differences in learning outcomes, and completion and satisfaction, among five staff roles including 274 aides (CNA, CMA), 180 nurses (LPN, RN), 52 administrators, 42 professional staff (social work, speech-, physical-, occupational-therapy), and 134 other non-care staff (dietary, custodial, transport). We compared changes in knowledge of effective person-centered communication, measured by (1) scores on the Changing Talk Scale (CHATS) and (2) ratings of video-based communication appropriateness, communication effectiveness, recognizing person-centered, and elderspeak communication. At baseline, aides and non-care staff had significantly lower CHATS scores than professional staff (p<.05). All roles demonstrated similar and significant knowledge gain with mean score increases ranging from 17.8-24.6 (p’s <.001). Similar patterns of changes were observed for communication ratings. The overall completion rate for enrolled staff was 82.6%, of whom only 6.2% reported dissatisfaction. The completion and satisfaction outcomes did not vary by role. These findings suggest that CHATO can be a useful education program for enhancing person-centered communication across different staff roles. Although baseline knowledge was higher in certain roles, the knowledge gains, completion rates, and satisfaction ratings did not vary by staff role. It is important to remember that all staff, not just direct care staff, benefit from education in nursing homes.

